# LMTK3 as a spatially regulated and stage-dependent biomarker in epithelial ovarian tumorigenesis

**DOI:** 10.1016/j.omton.2026.201199

**Published:** 2026-04-06

**Authors:** Ella Ittner, Lucas Werner, Hugo Swenson, Anna Linder, Karin Sundfeldt, Ghassan Saed, Per Karlsson, Toshima Z. Parris, Anikó Kovács, Khalil Helou

**Affiliations:** 1 Department of Oncology, Institute of Clinical Sciences, Sahlgrenska Academy, University of Gothenburg, Gothenburg, Sweden; 2 Sahlgrenska Center for Cancer Research, Sahlgrenska Academy, University of Gothenburg, Gothenburg, Sweden; 3 Department of Obstetrics and Gynecology, Institute of Clinical Sciences, Sahlgrenska Academy, University of Gothenburg, Gothenburg, Sweden; 4 Department of Obstetrics and Gynecology, Wayne State University School of Medicine, Detroit, MI, USA; 5 Department of Gynecologic Oncology, Karmanos Cancer Institute, Detroit, MI, USA; 6 Department of Obstetrics and Gynecology, University of Jordan School of Medicine, Amman, Jordan; 7 Region Västra Götaland, Sahlgrenska University Hospital, Department of Clinical Pathology, Gothenburg, Sweden

**Keywords:** MT: Regular Issue, lemur tyrosine kinase 3, lemur tail kinase 3, LMTK3, ovarian cancer, tumor initiation, tumor progression, prognostic biomarker, immunohistochemistry

## Abstract

Lemur tyrosine kinase 3 (LMTK3) has been implicated in cancer prognosis and progression. While its role in early-stage epithelial ovarian cancer (EOC) has been explored, the dynamics of its subcellular expression across the disease spectrum remain unclear. LMTK3 protein expression was assessed by immunohistochemistry on tissue microarrays comprising benign, borderline, and malignant ovarian tumors (*n* = 532). Nuclear and cytoplasmic staining were quantified via histochemical scores (H-scores) and analyzed in relation to histotype, disease stage, and overall survival. LMTK3 was broadly expressed across all tumor types, with predominant nuclear localization in benign and borderline lesions. Malignant tumors exhibited increased cytoplasmic expression and a stronger nuclear-cytoplasmic correlation (ρ = 0.73, *p* < 0.001), indicating altered spatial regulation during tumor progression. Within malignant EOC, LMTK3 expression patterns varied significantly by histotype and disease stage, reflecting context-dependent subcellular regulation. High nuclear expression was associated with better survival in early-stage EOC (adj. hazard ratio [HR] 0.33, *p* < 0.001), while cytoplasmic dominance suggested a favorable outcome in advanced-stage cases in multivariable analysis (HR = 0.53, *p* = 0.047). LMTK3 expression and subcellular localization evolve during ovarian tumorigenesis. These patterns carry stage-specific prognostic implications, supporting LMTK3 as a spatially resolved biomarker for EOC stratification.

## Graphical abstract

**Figure:**
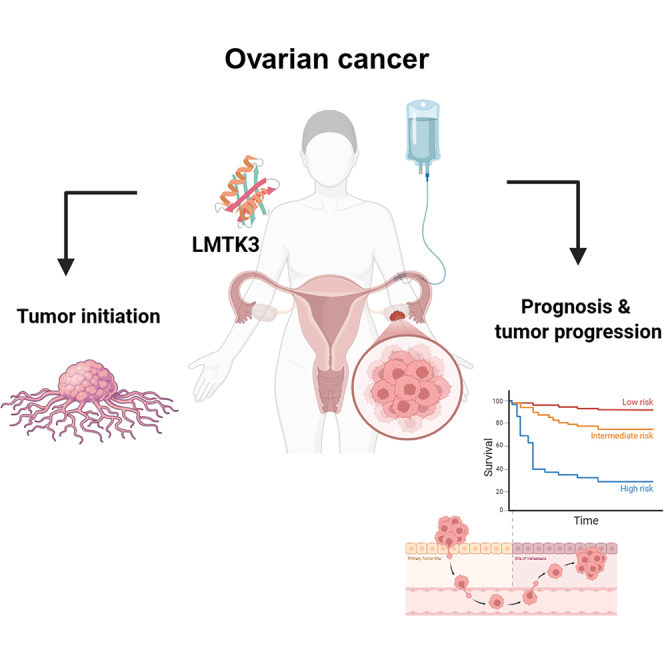

## Teaser

Subcellular localization of LMTK3 shows stage-dependent prognostic value in ovarian cancer. Nuclear and cytoplasmic expression patterns shift across benign, borderline, and malignant tumors, with opposite survival associations in early versus advanced disease, supporting spatially resolved LMTK3 assessment for risk stratification.

## Introduction

Ovarian cancer (OC) is the most lethal gynecologic malignancy, largely due to advanced-stage diagnosis. Epithelial OC (EOC) accounts for approximately 95% of malignant cases and represents a biologically diverse group of neoplasms.^1^ The majority of patients with EOC (∼75%) present with advanced-stage disease (stage III–IV), contributing to poor overall survival (OS) rates (∼31% 5-year survival vs. ∼51% in early-stage disease).^2^^,^^3^ EOC is substratified into distinct histopathological subgroups (so-called histotypes): high-grade serous (HGSC), low-grade serous (LGSC), endometrioid (EC), clear cell (CCC), and mucinous carcinoma (MC).^4^ . These subtypes differ significantly in terms of treatment response, recurrence patterns, and overall prognosis.^5^ Despite these distinct heterogeneous clinical characteristics, standard treatment strategies remain largely uniform, with debulking surgery followed by adjuvant chemotherapy.^6^ With advances made in recent years, such as the increasing use of neo-adjuvant chemotherapy or peritoneal therapy, the only alternative therapeutic option remains PARP (Poly (ADP-ribose) polymerase) inhibitors, which are available for patients with HGSC carrying a BRCA (breast cancer gene) mutation.^7^ Reliable clinical biomarkers are not available for these patient groups, as CA125 has failed to be sufficiently specific as a diagnostic screening tool and is more appropriately considered a monitoring tool.^8^ Prognostically, clinicopathological factors such as the Federation of Gynecology and Obstetrics (FIGO) stage and histotype are routinely used for risk assessment; however, no molecular prognostic biomarkers have been validated for clinical use in EOC.^9^ Although benign and borderline epithelial tumors are non-malignant, comparative analysis across the histologic spectrum may help clarify biomarker specificity for malignant transformation.^10^

Lemur tyrosine kinase 3 (LMTK3) belongs to the LMTK kinase family (LMTK1–3) and was initially studied in the context of neurodevelopment due to its high expression in the brain and links to disorders such as ADHD (attention deficit hyperactivity disorder) and autism.^11^^,^^12^ Structurally, LMTKs are large membrane-associated kinases characterized by an N-terminal kinase domain and a proline-rich C-terminal tail thought to mediate protein interactions.^13^ Although their functions remain incompletely defined, LMTK3 has emerged as a candidate biomarker and therapeutic target in cancer. In breast cancer, LMTK3 was identified through kinome-wide screening as a regulator of estrogen receptor α (Erα) and has been associated with tumor progression, endocrine resistance, and poor prognosis.^14^^,^^15^^,^^16^^,^^17^ Similar associations have been reported in gastric, bladder, thyroid, and lung cancers, where elevated LMTK3 expression or serum levels correlate with advanced disease and worse survival.^18^^,^^19^^,^^20^^,^^21^ Beyond these clinical associations, several studies have sought to characterize LMTK3 mechanistically and to understand how it contributes to tumorigenesis. Functional experiments in bladder and thyroid cancer models show that LMTK3 supports cell proliferation, migration, and survival, in part through modulation of mitogen-activated protein kinase (MAPK)-related signaling and apoptotic pathways.^19^^,^^21^ In breast cancer, LMTK3 also influences cytoskeletal dynamics and metastatic behavior, and its nuclear localization has been linked to transcriptional regulation of tumor-suppressive genes.^15^^,^^22^^,^^23^ Together, these findings suggest that LMTK3 may exert context-dependent oncogenic effects, with its subcellular distribution shaping distinct functional outputs. Building on this knowledge, we recently investigated the prognostic and biological role of LMTK3 in early-stage EOC, thereby showing that high nuclear LMTK3 expression was significantly associated with reduced OS and cytoplasmic dominance being unfavorable for prognosis.^24^ In the context of biomarker research, subcellular localization has been increasingly recognized as a critical determinant of protein function and clinical relevance. For kinases such as LMTK3, nuclear and cytoplasmic compartmentalization may reflect distinct roles in tumor progression and therapeutic response.^25^ Therefore, differentiating between nuclear and cytoplasmic expression is essential to fully evaluate the potential of LMTK3 as a biomarker in EOC. To expand upon our recent findings for LMTK3, the present study applies immunohistochemistry (IHC)-based histochemical score (H-score) quantification of nuclear and cytoplasmic LMTK3 expression across a large cohort containing early- and advanced-stage EOC, as well as benign and borderline epithelial ovarian tumors. This approach enables a more nuanced evaluation of LMTK3 as a potential biomarker by accounting for both expression levels and subcellular localization. Furthermore, we assessed the prognostic relevance of absolute expression and relative subcellular distribution in different disease stages, aiming to determine whether LMTK3 contributes to clinical risk stratification across the EOC spectrum.

## Results

### Cohort characteristics

The study cohort comprised 532 cases: 350 malignant EOC (65.8%), 59 borderline tumors (11.1%), and 123 benign lesions (23.1%; Table S1). The median age at diagnosis was 62 years (range: 16–88), with a median of 63 years for malignant cases (range: 22–88). Of the malignant cases, 199 (56.9%) were early-stage (I–II) and 151 (43.1%) were advanced-stage (III–IV), with stage I (37.7%) and stage III (38.0%) being most common. Age-based categories of menopausal status were: 13.1% premenopausal, 26.0% early postmenopausal, 28.3% intermediate postmenopausal, and 32.6% late postmenopausal. Histologically, malignant cases were predominantly HGSC (59.7%, *n* = 209), EC (14.6%, *n* = 51), CCC (12.0%, *n* = 42), MC (8.6%, *n* = 30), and LGSC (5.1%, *n* = 18; Table 1).

**Table 1:** Clinical, pathological, and LMTK3 expression characteristics of the malignant EOC cohort, stratified by histotype

	Study cohort: malignant EOC cases stratified by histotype						*p*-value
	Overall	CCC	EC	HGSC	LGSC	MC	
	350	42	51	209	18	30	
Age at Diagnosis (median [IQR])	63.00 [55.00, 73.00]	62.50 [54.00, 73.75]	61.00 [53.50, 74.00]	64.00 [55.00, 72.00]	58.00 [52.50, 68.50]	61.50 [52.75, 71.00]	**0.615**
Menopausal status							
Premenopausal (<50 years)	46 (13.1)	3 (7.1)	11 (21.6)	22 (10.5)	3 (16.7)	7 (23.3)	**0.301**
Early postmenopausal (50–59 years)	91 (26.0)	15 (35.7)	10 (19.6)	53 (25.4)	7 (38.9)	6 (20.0)	
Intermediate postmenopausal (60–69 years)	99 (28.3)	9 (21.4)	14 (27.5)	65 (31.1)	3 (16.7)	8 (26.7)	
Late postmenopausal (≤70 years)	114 (32.6)	15 (35.7)	16 (31.4)	69 (33.0)	5 (27.8)	9 (30.0)	
Stage							
I	132 (37.7)	28 (66.7)	32 (62.7)	47 (22.5)	4 (22.2)	21 (70.0)	**<0.001**
II	67 (19.1)	6 (14.3)	13 (25.5)	37 (17.7)	4 (22.2)	7 (23.3)	
III	133 (38.0)	8 (19.0)	5 (9.8)	109 (52.2)	10 (55.6)	1 (3.3)	
IV	18 (5.1)	0 (0.0)	1 (2.0)	16 (7.7)	0 (0.0)	1 (3.3)	
Disease stage group							
Early-stage (I/II)	199 (56.9)	34 (81.0)	45 (88.2)	84 (40.2)	8 (44.4)	28 (93.3)	**<0.001**
Advanced-stage (III/IV)	151 (43.1)	8 (19.0)	6 (11.8)	125 (59.8)	10 (55.6)	2 (6.7)	
Initial treatment response							
complete response	98 (28.0)	4 (9.5)	4 (7.8)	81 (38.8)	9 (50.0)	0 (0.0)	**<0.001**
partial response	24 (6.9)	1 (2.4)	0 (0.0)	23 (11.0)	0 (0.0)	0 (0.0)	
progressive disease	7 (2.0)	2 (4.8)	0 (0.0)	3 (1.4)	1 (5.6)	1 (3.3)	
stable disease	1 (0.3)	0 (0.0)	0 (0.0)	1 (0.5)	0 (0.0)	0 (0.0)	
Not available	220 (62.9)	35 (83.3)	47 (92.2)	101 (48.3)	8 (44.4)	29 (96.7)	
Debulking surgery status							
No	82 (23.4)	3 (7.1)	1 (2.0)	73 (34.9)	4 (22.2)	1 (3.3)	**<0.001**
Yes	244 (69.7)	37 (88.1)	46 (90.2)	128 (61.2)	8 (44.4)	25 (83.3)	
Not available	24 (6.9)	2 (4.8)	4 (7.8)	8 (3.8)	6 (33.3)	4 (13.3)	
Overall survival status							
0–2 years	49 (14.0)	10 (23.8)	3 (5.9)	29 (13.9)	0 (0.0)	7 (23.3)	**<0.001**
2–5 years	106 (30.3)	12 (28.6)	9 (17.6)	80 (38.3)	3 (16.7)	2 (6.7)	
5–10 years	96 (27.4)	7 (16.7)	11 (1.6)	62 (29.7)	9 (50.0)	7 (23.3)	
>10 years	97 (27.7)	13 (31.0)	28 (54.9)	36 (17.2)	6 (33.3)	14 (46.7)	
Not available	2 (0.6)	0 (0.0)	0 (0.0)	2 (1.0)	0 (0.0)	0 (0.0)	
Cause of Death							
Ovarian cancer	118 (33.7)	22 (52.4)	7 (13.7)	82 (39.2)	2 (11.1)	5 (16.7)	**<0.001**
Other cancer	20 (5.7)	2 (4.8)	5 (9.8)	8 (3.8)	0 (0.0)	5 (16.7)	
Other disease	35 (10.0)	6 (14.3)	10 (19.6)	10 (4.8)	0 (0.0)	9 (30.0)	
Treatment complication	1 (0.3)	0 (0.0)	0 (0.0)	1 (0.5)	0 (0.0)	0 (0.0)	
Alive	110 (31.4)	9 (21.4)	26 (51.0)	52 (24.9)	14 (77.8)	9 (30.0)	
Not available	66 (18.9)	3 (7.1)	3 (5.9)	56 (26.8)	2 (11.1)	2 (6.7)	
Nuclear staining intensity							
Negative	3 (0.9)	1 (2.4)	1 (2.0)	1 (0.5)	0 (0.0)	0 (0.0)	**0.007**
Low	40 (11.4)	11 (26.2)	4 (7.8)	23 (11.0)	0 (0.0)	2 (6.7)	
Moderate	73 (20.9)	14 (33.3)	11 (21.6)	42 (20.1)	4 (22.2)	2 (6.7)	
High	234 (66.9)	16 (38.1)	35 (68.6)	143 (68.4)	14 (77.8)	26 (86.7)	
Nuclear positivity (% of cells)							
None (0%)	3 (0.9)	1 (2.4)	1 (2.0)	1 (0.5)	0 (0.0)	0 (0.0)	**0.379**
Low (1–10%)	4 (1.1)	2 (4.8)	0 (0.0)	1 (0.5)	0 (0.0)	1 (3.3)	
Moderate (11–50%)	19 (5.4)	2 (4.8)	1 (2.0)	12 (5.7)	2 (11.1)	2 (6.7)	
High (51–100%)	324 (92.6)	37 (88.1)	49 (96.1)	195 (93.3)	16 (88.9)	27 (90.0)	
Nuclear H-score (median [IQR])	300.00 [185.00, 300.00]	200.00 [100.00, 300.00]	300.00 [190.00, 300.00]	300.00 [180.00, 300.00]	285.00 [200.00, 300.00]	300.00 [300.00, 300.00]	**0.002**
Cytoplasmic staining intensity							
Negative	3 (0.9)	0 (0.0)	0 (0.0)	2 (1.0)	1 (5.6)	0 (0.0)	**0.004**
Low	44 (12.6)	10 (23.8)	2 (3.9)	26 (12.4)	2 (11.1)	4 (13.3)	
Moderate	113 (32.3)	23 (54.8)	14 (27.5)	60 (28.7)	6 (33.3)	10 (33.3)	
High	185 (52.9)	8 (19.0)	35 (68.6)	117 (56.0)	9 (50.0)	16 (53.3)	
Not available	5 (1.4)	1 (2.4)	0 (0.0)	4 (1.9)	0 (0.0)	0 (0.0)	
Cytoplasmic positivity (% of cells)							
None (0%)	3 (0.9)	0 (0.0)	0 (0.0)	2 (1.0)	1 (5.6)	0 (0.0)	**0.64**
Moderate (11–50%)	4 (1.1)	1 (2.4)	0 (0.0)	3 (1.4)	0 (0.0)	0 (0.0)	
High (51–100%)	338 (96.6)	40 (95.2)	51 (100.0)	200 (95.7)	17 (94.4)	30 (100.0)	
Not available	5 (1.4)	1 (2.4)	0 (0.0)	4 (1.9)	0 (0.0)	0 (0.0)	
Cytoplasmic H-score (median [IQR])	300.00 [200.00, 300.00]	200.00 [170.00, 200.00]	300.00 [200.00, 300.00]	300.00 [200.00, 300.00]	205.00 [200.00, 300.00]	300.00 [200.00, 300.00]	**<0.001**
LMTK3 localization pattern							
Nuclear-dominant (N > C)	67 (19.1)	12 (28.6)	1 (2.0)	40 (19.1)	4 (22.2)	10 (33.3)	**<0.001**
balanced (C = N)	211 (60.3)	14 (33.3)	41 (80.4)	127 (60.8)	13 (72.2)	16 (53.3)	
Cytoplasmic-dominant (C > N)	68 (19.4)	16 (38.1)	9 (17.6)	38 (18.2)	1 (5.6)	4 (13.3)	
Not available	4 (1.1)	0 (0.0)	0 (0.0)	4 (1.9)	0 (0.0)	0 (0.0)	

Categorical variables were compared using Chi-squared or Fisher’s exact test, as appropriate. Abbreviations are as follows: CCC, clear cell carcinoma; EC, endometrioid carcinoma; EOC, epithelial ovarian cancer; HGSC, high-grade serous carcinoma; LGSC, low-grade serous carcinoma; LMTK3, lemur tyrosine kinase 3; MC, mucinous carcinoma.

Data on radical surgery, defined as complete macroscopic tumor debulking (no residual disease), were available for 326 cases (93.1%). Of these, 74.8% underwent radical surgery, while 25.2% had visible residual disease postoperatively. Information on initial treatment response to chemotherapy was available for 300 of 350 malignant EOC cases (85.7%), of which 87.7% achieved a complete response, 8.7% had a partial response, 3.3% experienced progressive disease, and 0.3% had stable disease. Median OS was 6.2 years and disease-specific survival (DSS) was 3.4 years. The estimated 5-year OS in the full malignant cohort was 56.1% (95% confidence interval [CI]: 51.1%–61.6%). Stratified by disease stage, 5-year OS was 67.3% (95% CI: 61.1–74.2%) in early-stage and 39.6% (95% CI: 32.5–48.3%) in advanced-stage patients.

### LMTK3 subcellular localization shifts along the spectrum of ovarian tumorigenesis

To explore LMTK3 protein localization and abundance across the histopathologic spectrum of ovarian tumors, H-scores representing nuclear and cytoplasmic immunostaining were compared among benign, borderline, and malignant epithelial ovarian lesions. LMTK3 was detectable in nearly all samples included in the analysis. Nuclear expression (defined as H-score > 0) was observed in 100% of benign (*n* = 104/104) and borderline tumors (*n* = 46/46) and in 99.1% of malignant tumors (*n* = 343/346). Cytoplasmic expression (also defined as H-score > 0) was equally prevalent: 96.2% of benign tumors (*n* = 100/104), 100% of borderline tumors (*n* = 46/59), and 99.1% of malignant tumors (*n* = 343/346) showed positive cytoplasmic staining. Representative examples of nuclear-dominant, cytoplasmic-dominant, and balanced LMTK3 immunostaining patterns are shown in Figure S1.

**Figure 1:**
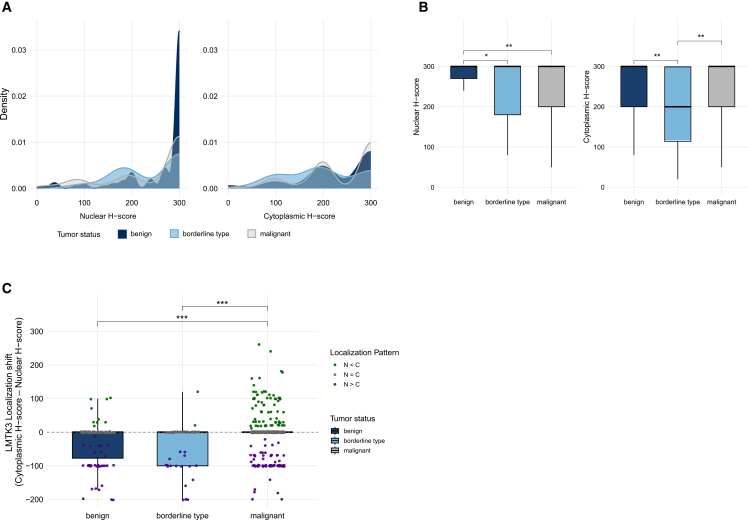
Tumor status-dependent variation in LMTK3 expression and localization (A) Density plots of nuclear and cytoplasmic LMTK3 H-scores across benign, borderline, and malignant tumors demonstrate the distribution and range of subcellular expression levels. (B) Boxplots comparing nuclear and cytoplasmic LMTK3 H-scores by tumor type highlight consistently high nuclear expression and variable cytoplasmic expression, particularly in borderline lesions. (C) Localization shift scores and subcellular pattern classifications illustrate a transition from nuclear-dominant localization in benign and borderline tumors to increased cytoplasmic enrichment in malignant samples. Statistical significance was assessed using Kruskal-Wallis tests followed by pairwise Wilcoxon rank-sum tests with Benjamini-Hochberg correction; significance of the adjusted *p* values indicated by asterisks. ∗*p* < 0.05, ∗∗*p* < 0.01, ∗∗∗*p* < 0.001, and ∗∗∗∗*p* < 0.0001. Abbreviations are as follows: LMTK3, lemur tyrosine kinase 3.

Boxplots and density plots revealed distinct nuclear and cytoplasmic LMTK3 expression profiles across tumor types (Figures 1A and 1B). Nuclear H-scores were consistently high in both benign (median: 300; IQR (interquartile range): 270–300; range: 5–300) and malignant tumors (median: 300; IQR: 240–300; range: 0–300), whereas borderline tumors displayed slightly lower expression levels and greater variability (median: 270; IQR: 180–300; range: 10–300; *p* = 0.021). Significantly lower nuclear expression was found in borderline tumors compared with both benign (p-adj = 0.042) and malignant tumors (p-adj = 0.024). Cytoplasmic H-scores followed a similar pattern: benign and malignant tumors exhibited high expression levels (both medians: 300; IQRs: 250–300; range: 0–300), while borderline tumors showed markedly reduced and heterogeneous expression (median: 200; IQR: 100–285; range: 0–300). These group differences were significant (*p* = 0.012), with post hoc comparisons showing significantly lower cytoplasmic expression in borderline tumors compared with both the benign and malignant groups (p-adj = 0.012 for both).

Differences in LMTK3 subcellular localization across EOC tumor types were examined using both the localization shift score and the subcellular LMTK3 pattern categories (Figure 1C). Localization shift scores quantify both the direction and extent of LMTK3 subcellular localization dominance, with positive values indicating cytoplasmic enrichment and negative values indicating nuclear predominance. Benign and borderline tumors showed mostly negative scores, reflecting nuclear-dominant localization, whereas malignant tumors exhibited more positive scores, consistent with increased cytoplasmic presence of LMTK3. Scores differed significantly across the three EOC tumor types (*p* < 0.001), with malignant tumors showing significantly higher scores than both benign (p-adj <0.001) and borderline tumors (p-adj <0.001); no significant difference was observed between benign and borderline tumors (p-adj = 0.416). The distribution of subcellular LMTK3 patterns also varied significantly across tumor types (*p* < 0.001), supporting a shift in compartmental localization that may accompany malignant transformation.

### LMTK3 expression differs across EOC histotypes and disease stages

**Figure 2:**
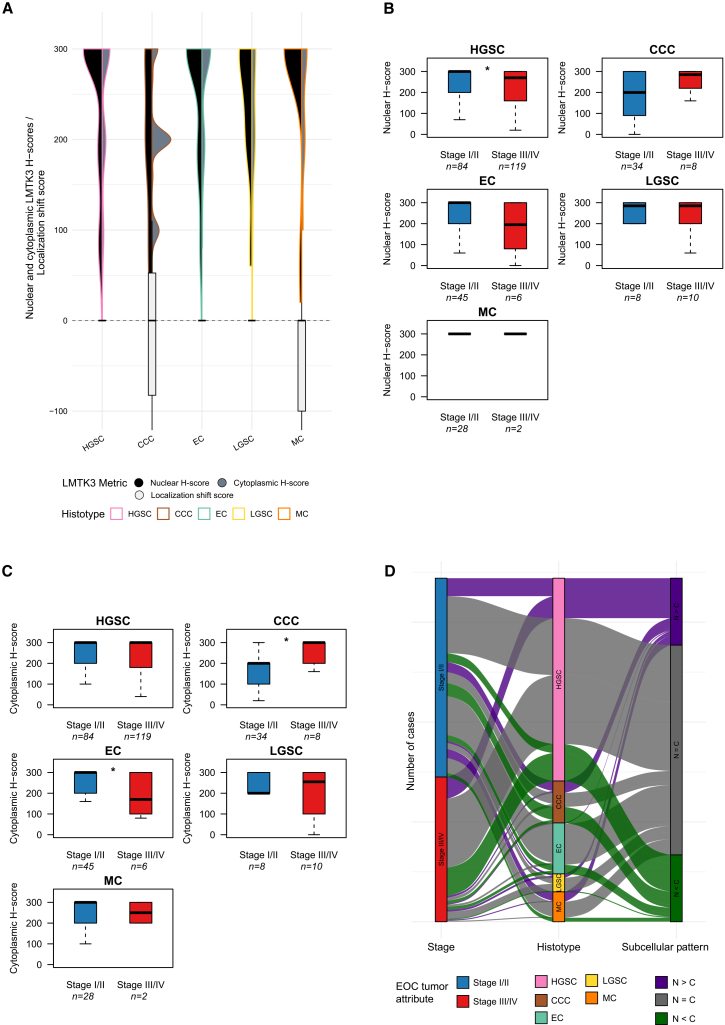
LMTK3 compartmental expression in malignant EOC: histotype and stage effects (A) Violin plots visualizing nuclear and cytoplasmic LMTK3 H-scores across the five main EOC histotypes (HGSC, CCC, EC, LGSC, and MC), accompanied by localization shift scores summarizing compartmental dominance per histotype. (B) Stratified boxplots comparing nuclear LMTK3 expression between early-stage (I/II) and advanced-stage (III/IV) tumors across histotypes. (C) Stage-stratified cytoplasmic LMTK3 expression revealing histotype-specific patterns. (D) Alluvial plot illustrating the distribution of LMTK3 subcellular expression patterns across histotype and disease stage. Distinct subcellular localization patterns emerge among histotypes, particularly in early-stage disease. Asterisks in (B) and (C) indicate statistical significance based on Wilcoxon rank-sum tests with Benjamini-Hochberg correction. ∗*p* < 0.05, ∗∗*p* < 0.01, ∗∗∗*p* < 0.001, and ∗∗∗∗*p* < 0.0001. Abbreviations are as follows: CCC, clear cell carcinoma; EC, endometrioid carcinoma; EOC, epithelial ovarian cancer; HGSC, high-grade serous carcinoma; LGSC, low-grade serous carcinoma; LMTK3, lemur tyrosine kinase 3; MC, mucinous carcinoma.

Subsequent analyses focused on malignant EOC cases to examine histotype- and stage-specific LMTK3 expression using H-scores. Nuclear expression was significantly lower in advanced-stage tumors (median: 270, *p* = 0.014), while cytoplasmic expression did not differ (median: 300, *p* = 0.501). LMTK3 expression varied significantly across histotypes (nuclear: *p* = 0.002, cytoplasmic: *p* < 0.001). The localization shift score showed a non-significant trend (*p* = 0.061; Figure 2A). Stratification by stage revealed decreased nuclear expression in advanced-stage HGSC (p-adj = 0.015), but no stage-related changes in other histotypes (Figure 2B). Cytoplasmic expression in EC decreased in advanced-stage (p-adj = 0.041), while CCCs showed increased cytoplasmic LMTK3 from early to advanced stage (p-adj = 0.025) (Figure 2C).

To explore the distribution of subcellular LMTK3 expression patterns in relation to both histotype and disease stage, we visualized the cohort using an alluvial plot (Figure 2D). Subcellular patterns varied significantly across histotypes (Chi-squared *p* < 0.001; Fisher’s exact *p* < 0.001), with pairwise comparisons indicating a relative enrichment of cytoplasmic-dominant expression in CCCs (p-adj <0.001–0.005). In contrast, no statistically significant differences in overall subcellular pattern distribution were observed across early- vs. advanced-stage tumors (Fisher’s *t* test *p* = 0.160). However, when stratified by stage, early-stage tumors showed a significant difference in subcellular pattern across histotypes (Fisher’s exact *p* < 0.001), which was not seen in advanced-stage tumors (*p* = 0.540). For example, early-stage CCCs and MCs showed increased proportions of cytoplasmic-dominant LMTK3, whereas ECs primarily exhibited a balanced pattern (86.7%).

### Coordinated subcellular LMTK3 expression across EOC histotypes and stages

**Figure 3:**
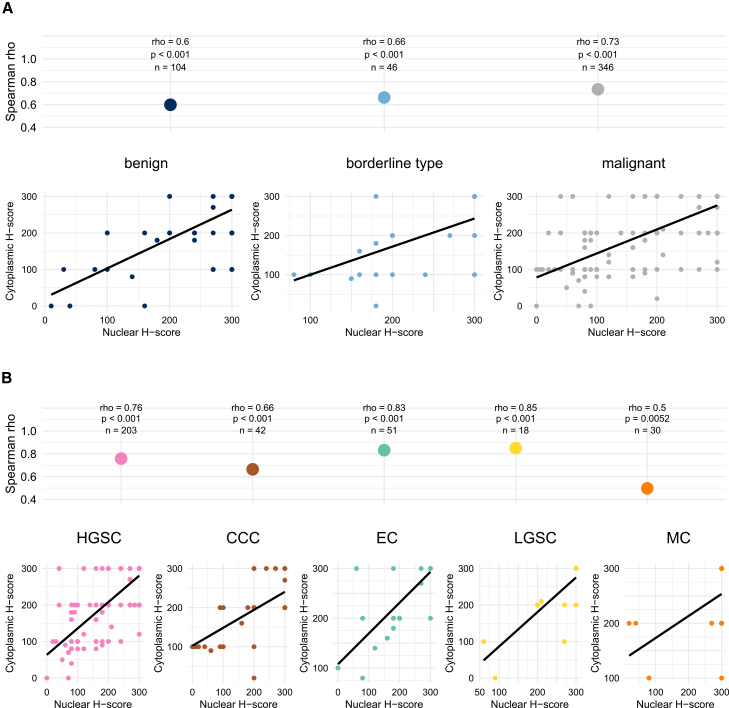
Correlation between nuclear and cytoplasmic LMTK3 expression across EOC tumor groups (A) Spearman correlation coefficients (ρ) between nuclear and cytoplasmic LMTK3 H-scores across the EOC tumor spectrum (benign, borderline, and malignant). Boxplots summarize the correlation strength; individual dots represent the observed correlations, with annotated ρ, *p* value, and sample size (*n*) for each group. Scatterplots below visualize the subcellular correlation for each group. Linear regression lines are shown in black. (B) The same analysis for the malignant subcohort stratified by histotype (HGSC, LGSC, CCC, EC, and MC). Abbreviations are as follows: CCC, clear cell carcinoma; EC, endometrioid carcinoma; EOC, Epithelial-ovarian cancer; HGSC, high-grade serous carcinoma; LGSC, low-grade serous carcinoma; LMTK3, lemur tyrosine kinase 3; MC, mucinous carcinoma.

To analytically conclude the sub-compartment relationship for LMTK3 in EOC, correlation analysis between nuclear and cytoplasmic LMTK3 expression across the full tumor spectrum was implemented. Spearman correlation analysis revealed increasing correlation strength from benign (ρ = 0.60) to borderline (ρ = 0.66) to malignant tumors (ρ = 0.73), with all associations statistically significant (*p* < 0.001). These findings support coordinated subcellular localization of LMTK3 across EOC, with a more pronounced correlation in more aggressive disease (Figure 3A). Within the malignant subset, nuclear-cytoplasmic expression remained strongly correlated in both early-stage (ρ = 0.71, *p* < 0.001) and advanced-stage (ρ = 0.76, *p* < 0.001) tumors. This association held across histotypes (Figure 3B), though with some variability: correlations ranged from moderate in MC (ρ = 0.50, *p* = 0.005) to very strong in LGSC (ρ = 0.85, *p* < 0.001). Further stratification by histotype and stage (Figure S2) showed that most histotypes—HGSC, EC, and LGSC—maintained strong correlations across both early and advanced stages. In EC, despite a strong effect size in advanced-stage tumors (ρ = 0.81), significance was not reached (*p* = 0.051), likely due to limited sample size (*n* = 6). Notably, in CCC, the early-stage correlation was significant (ρ = 0.67, *p* < 0.001) but was lost in advanced-stage tumors (ρ = 0.10, *p* = 0.81, *n* = 8), suggesting a potential breakdown of subcellular coordination in advanced CCC. In MC, data were only available for early-stage tumors, which showed a moderate correlation (ρ = 0.51, *p* = 0.005).

### Exploratory transcriptomic analyses do not reveal a dominant LMTK3-associated program

To assess whether the stage- and compartment-specific behavior of LMTK3 observed at the protein level is reflected at the transcriptional level, we performed exploratory analyses using TCGA-OV (The Cancer Genome Atlas Ovarian Serous Cystadenocarcinoma) transcriptomes. Global co-expression patterns were modest, indicating that LMTK3 is not embedded in a strong or unified transcriptional module. Using TCGA OC (TCGA-OV) transcriptomic data in GEPIA2, LMTK3 mRNA expression was evaluated across clinical stages. No statistically significant differences in LMTK3 expression were observed between stages at the transcriptomic level (ANOVA *p* = 0.0587; Figure S3A). Unbiased Gene Ontology (GO) enrichment of the top-correlated LMTK3 transcripts yielded broad categories related to small GTPase (guanosine triphosphatase) activity, endocytic vesicles, recycling endosomes, and focal adhesions, suggesting associations with membrane trafficking and adhesion-related processes but without evidence of a dominant signaling program.

Targeted analyses of biologically relevant oncogenic pathways, as well as components of our previously proposed mechanistic model, showed positive correlations between LMTK3 and integrin-related genes (ITGAV, ITGB1) and proliferation markers (MKI67, CCNB1), along with weaker or inverse correlations with apoptotic mediators (CASP3, CASP9, BAX, BCL2) and EMT (epithelial-mesenchymal transition) -related genes (SNAI1, VIM). Thus, although LMTK3 shows transcriptional links to adhesion and survival pathways, the weak overall correlations suggest that bulk mRNA patterns provide only limited insight into its functional role, which may be better captured at the protein and subcellular levels (Figures S3B–S3O).

### Prognostic relevance of absolute LMTK3 expression in early- and advanced-stage EOC

**Figure 4:**
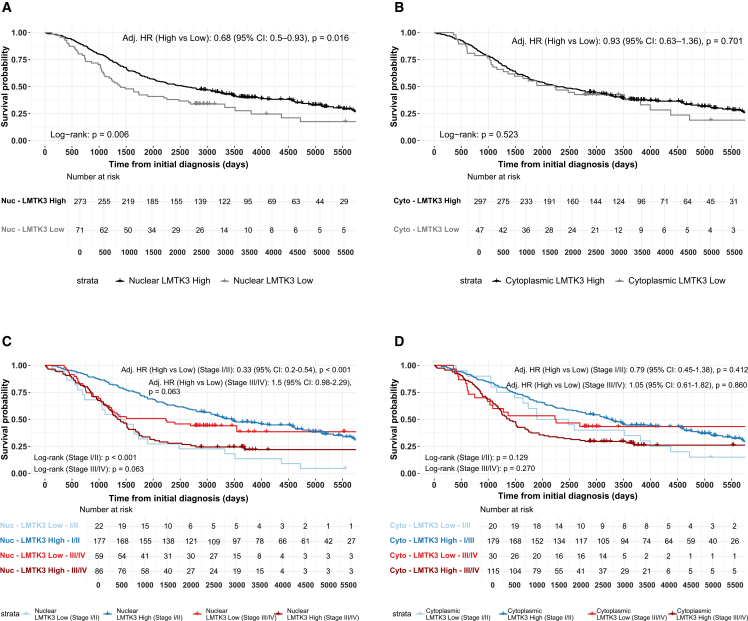
Survival impact of absolute LMTK3 expression in nucleus and cytoplasm across complete, early-, and advanced-stage EOC Kaplan-Meier overall survival (OS) curves, with a 15-year truncation, are shown for high vs. low absolute LMTK3 expression, based on optimized H-score cutoffs. (A) Nuclear expression across the complete EOC cohort. (B) Cytoplasmic expression in the same cohort. (C) Nuclear expression stratified by early-stage (stage I/II) and advanced-stage (stage III/IV) disease. (D) Cytoplasmic expression stratified by disease stage. Hazard ratios (HRs) and adjusted *p* values from multivariable Cox regression models (adjusted for age group, histotype, and stage, where appropriate) are displayed within each panel. Statistical differences between survival curves were evaluated using log-rank testing, also indicated in each subplot. Abbreviations are as follows: EOC, epithelial ovarian cancer; LMTK3, lemur tyrosine kinase 3.

In the complete EOC cohort, low nuclear LMTK3 expression was significantly associated with worse OS (Figure 4). In univariable Cox regression, patients with high nuclear expression had a hazard ratio (HR) of 0.65 (95% CI: 0.48–0.89, *p* = 0.006). This association remained significant in the multivariable analysis adjusting for age (grouped into menopausal-status proxies), stage, and histotype (adj. HR = 0.68, 95% CI: 0.50–0.93, *p* = 0.016). In contrast, cytoplasmic LMTK3 expression was not prognostic in either univariable (HR = 0.89, 95% CI: 0.61–1.29, *p* = 0.523) or multivariable models (adj. HR = 0.93, 95% CI: 0.63–1.36, *p* = 0.701).

To assess stage-specific effects, the cohort was stratified into early- and advanced-stage disease. In early-stage EOC, high nuclear LMTK3 expression was associated with markedly improved OS (adj. HR = 0.33, 95% CI: 0.20–0.54, *p* < 0.001), supporting previous findings using non-H-score-based classification. Among early-stage patients, the subgroup with low nuclear LMTK3 expression was small (*n* = 22) but showed very low absolute H-scores (median 75; IQR 25–80), confirming a true low-expression phenotype. Their age distribution was comparable to that of early-stage patients with high expression (median, 62 years [IQR 56–79] vs. 64 years [IQR 54–75]). Histologically, early-stage low-expression tumors were enriched for CCC (40.9%) and had fewer HGSC tumors (27.3%) compared with the high-expression group (14.1% and 44.1%, respectively). Outcomes in this subgroup were poor: only 1/22 (4.5%) patients were alive at last follow-up, with 14/22 (63.6%) deaths attributed to EOC. In contrast, among early-stage patients with high nuclear LMTK3 expression (*n* = 177), 64/177 (36.2%) were alive at last follow-up, with 61/177 (34.5%) OC-related deaths. These findings indicate that early-stage low nuclear LMTK3 expression identifies a biologically distinct and clinically aggressive subgroup within the cohort.

Cytoplasmic expression showed no significant association in multivariable models (adj. HR = 1.26, 95% CI: 0.72–2.20, *p* = 0.412). In advanced-stage EOC, high nuclear expression lost significance but indicated worse prognosis (adj. HR = 1.5, 95% CI: 0.98–2.29, *p* = 0.063). Cytoplasmic expression remained non-significant (adj. HR = 1.05, 95% CI: 0.61–1.82, *p* = 0.860), suggesting limited prognostic relevance in this subgroup. To visualize the differential prognostic effects of LMTK3 localization, KM (Kaplan-Meier) survival curves were generated (Figures 4A–4D). In the complete cohort, low nuclear expression predicted poorer OS, whereas cytoplasmic expression showed no prognostic relevance. Stage-stratified analyses confirmed this pattern in early-stage EOC, while in advanced-stage disease, the directionality of nuclear prognostic impact was reversed. Cytoplasmic expression remained non-significant across all analyses.

Additionally, analyses for DSS were performed, which largely mirrored the results for OS (Figure S4). In the complete cohort, low nuclear expression was linked to worse DSS in the univariable analysis (HR = 1.68, 95% CI: 1.01–2.78, *p* = 0.045), though the adjusted model was only close to significant (adj. HR = 1.58, 95% CI: 0.95–2.63, *p* = 0.081). No cytoplasmic association was observed (adj. HR = 1.01, 95% CI: 0.62–1.65, *p* = 0.969). In early-stage EOC, low nuclear expression was a strong negative prognostic marker (adj. HR = 2.93, 95% CI: 1.59–5.40, *p* < 0.001). Interestingly, low cytoplasmic expression showed an association with worse DSS in univariable analysis (HR = 2.02, 95% CI: 1.11–3.68, *p* = 0.021) but was not significant after adjustment (adj. HR = 1.61, 95% CI: 0.84–3.07, *p* = 0.152). In advanced-stage EOC, nuclear expression remained associated with better DSS (adj. HR = 0.39, 95% CI: 0.19–0.79, *p* = 0.009), reinforcing the reversal in prognostic directionality. Cytoplasmic expression again did not show significant prognostic value in the multivariable analysis (Table S2).

### Internal assessment of robustness of nuclear LMTK3 prognostic effects

To assess the robustness of these findings, we performed two complementary internal validation approaches: split-sample validation and non-parametric bootstrap resampling (1,000 iterations). In the complete cohort, random 2:1 derivation-validation splitting reproduced the prognostic effect of nuclear LMTK3, with a consistent direction of association in the validation set (C-index = 0.59). In early-stage EOC, the prognostic effect of low nuclear LMTK3 was highly stable: the validation subset retained a strong association with survival (HR = 3.15, 95% CI: 1.93–5.16; C-index = 0.71). Bootstrap resampling confirmed this robustness, yielding a narrow HR distribution (median 0.31, 95% bootstrap interval 0.16–0.53) and a high probability that low nuclear expression is associated with worse outcome (P[HR < 1] = 1.00).

In contrast, as expected, prognostic effects in advanced-stage EOC were weaker and unstable. Split-sample validation showed only a modest and non-reproducible association (validation HR = 1.06, *p* = 0.21; C-index = 0.62). Bootstrap results supported this instability, with a wide HR distribution centered near 1.5 (95% bootstrap interval 0.93–2.53) and only 5% of iterations indicating HR < 1. Cytoplasmic expression remained non-prognostic in both validation approaches across all stages. Together, these internal validation analyses demonstrate that the prognostic utility of LMTK3 is strong and reproducible in early-stage EOC (Figure S5A).

### Transcript-level analysis of LMTK3 expression

To assess whether the robust protein-based prognostic effects of LMTK3 were reflected at the transcript level, we examined bulk LMTK3 mRNA expression in an independent RNA sequencing (RNA-seq) cohort and in a matched early-stage subset with available IHC. In contrast to the strong and reproducible associations observed for nuclear LMTK3 protein, transcript-level expression showed no robust prognostic signal. In the matched early-stage cohort (*n* = 78), higher LMTK3 mRNA expression was associated with a modest improvement in OS when modeled as a continuous variable (HR per standard deviation increase = 0.79, 95% CI: 0.61–1.03; *p* = 0.066), but this association did not reach conventional statistical significance and was not stable in joint models including nuclear protein expression. Moreover, LMTK3 mRNA levels did not correlate with nuclear (Spearman ρ = −0.12, *p* = 0.31) or cytoplasmic (ρ = −0.10, *p* = 0.38) protein H-scores, indicating that transcript abundance does not explain the localization-dependent prognostic effects observed at the protein level (see Table S3; Figures S5B–S5D).

### Subcellular distribution of LMTK3 reveals opposing stage-specific prognostic effects

**Figure 5:**
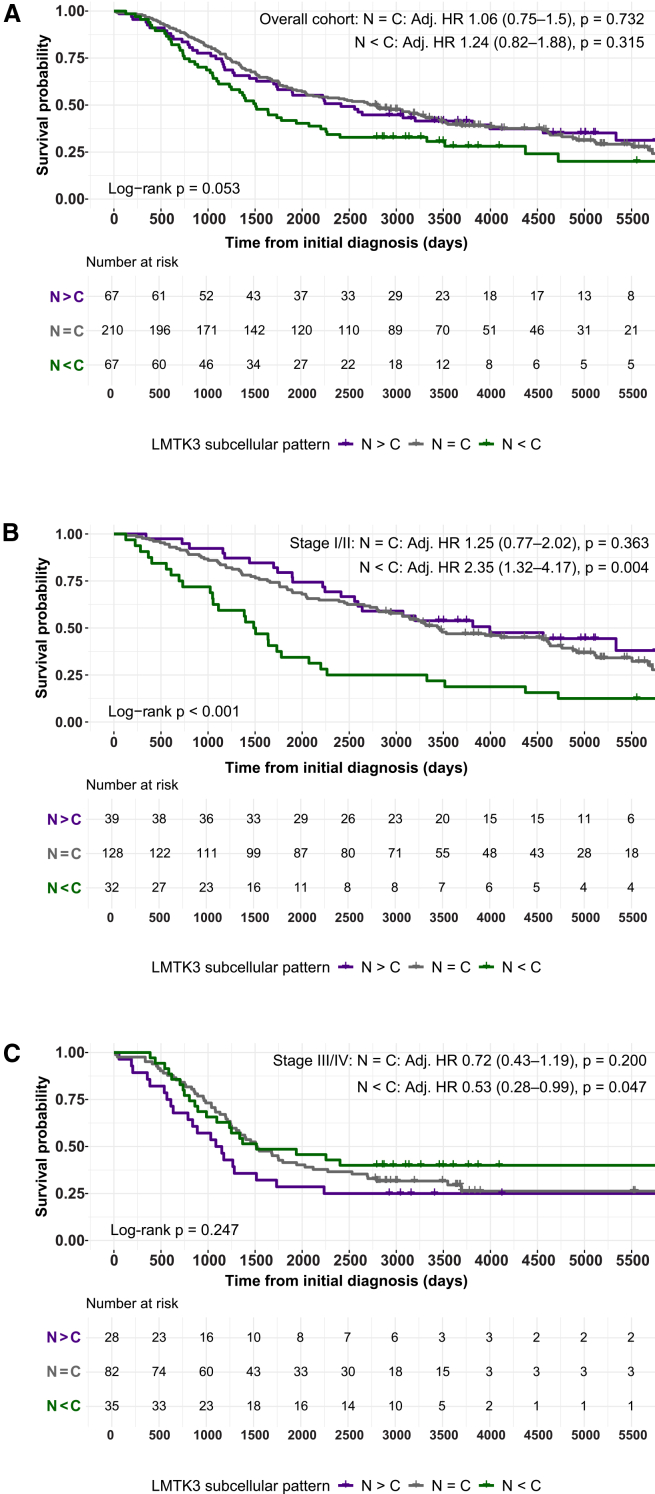
Prognostic impact of subcellular LMTK3 localization pattern across disease stages in EOC Kaplan-Meier survival curves stratified by LMTK3 localization pattern: nuclear-dominant (N > C), balanced (N = C), and cytoplasmic-dominant (N < C). (A) Complete malignant cohort. (B) Early-stage subcohort (stage I–II). (C) Advanced-stage subcohort (stage III–IV). Multivariable Cox models were adjusted for age group and histotype; nuclear-dominant expression served as the reference. Additionally, statistical differences between survival curves were evaluated using log-rank testing, also indicated in each subplot. Abbreviations are as follows: EOC, epithelial ovarian cancer; LMTK3, lemur tyrosine kinase 3.

To explore the biological impact of LMTK3 localization, we analyzed the subcellular dominance of expression based on H-scores, reflecting sub-compartmentalized LMTK3 expression. In the complete EOC cohort, subcellular dominance was not significantly associated with survival (multivariable: *p* > 0.3; Figure 5A). However, stage-stratified analyses revealed a distinct divergent prognostic impact of LMTK3. In early-stage EOC, cytoplasmic-dominant expression (N < C) was significantly associated with worse OS (adj. HR = 2.35, 95% CI: 1.32–4.17, *p* = 0.004), and this pattern held for DSS as well (adj. HR = 2.50, 95% CI: 1.18–5.30, *p* = 0.017; Figure 5B). These findings align with prior non-H-score-based analyses and newly confirm the association for DSS. In contrast to early-stage EOC, in advanced-stage EOC, cytoplasmic-dominant expression was associated with better outcomes. For OS, the multivariable model showed a protective association (adj. HR = 0.53, 95% CI: 0.28–0.99, *p* = 0.047), while the DSS analysis confirmed this pattern (adj. HR = 0.30, 95% CI: 0.09–0.99, *p* = 0.049; Figure 5C). Balanced expression (N = C) did not significantly differ from nuclear-dominant expression in either stage. These findings were formally supported by a Cox proportional hazards model including an interaction term between subcellular localization and disease stage (early vs. advanced), which confirmed a statistically significant interaction (*p* = 0.002), indicating that the prognostic impact of LMTK3 localization is stage-dependent and biologically distinct.

## Discussion

Using large-scale IHC on a comprehensive tissue microarray (TMA)-based ovarian tumor cohort, we present the first systematic analysis of LMTK3 protein expression and subcellular localization across the full spectrum of epithelial ovarian tumors, including benign, borderline, and malignant epithelial ovarian tumors, with stratification by histotype and stage. Building on our previous work in early-stage EOC, this study expands the understanding of LMTK3 by investigating its expression dynamics and prognostic relevance in advanced-stage disease, a context that remains largely unexplored. We show that LMTK3 localization shifts from nuclear dominance in benign and borderline tumors toward increased cytoplasmic presence in malignant lesions, with clear differences across histotypes. Furthermore, we identify that nuclear LMTK3 expression serves as a prognostic marker in a stage-dependent manner, associated with unfavorable outcomes in early-stage EOC but favorable survival in advanced-stage disease. These findings align with our earlier analysis, which initially identified nuclear LMTK3 expression as a prognostic biomarker for early-stage EOC.

LMTK3 has been reported to be overexpressed in several solid malignancies, including breast, colorectal, and thyroid cancer,^18^^,^^21^^,^^26^ but expression studies in non-malignant tissues are rare. Zhang et al. reported elevated soluble LMTK3 (sLMTK3) levels in the serum of patients with NSCLC (non-small-cell lung cancer), with a progressive increase from healthy controls to benign lung lesions and highest levels in malignancy, suggesting a stepwise upregulation during tumorigenesis.^27^ Similarly, IHC analysis of brain tumor specimens revealed detectable LMTK3 in normal brain and benign meningiomas, with increased expression in high-grade gliomas.^28^ These findings support a broader physiological role of LMTK3 beyond overt malignancy. Our study extends this perspective to epithelial ovarian tumors, where LMTK3 was found to be near-universally expressed in benign, borderline, and malignant lesions. Borderline tumors exhibited the most heterogeneous and attenuated expression levels, suggesting transitional subcellular regulation during tumor initiation. Beyond documenting absolute expression, we provide a refined spatial characterization, revealing a shift from predominantly nuclear localization in benign and borderline tumors to increased cytoplasmic expression in malignancy. This shift in subcellular distribution may not only reflect but also contribute to evolving biological states along the tumor spectrum.

In malignant EOC, LMTK3 remained robustly expressed in both nuclear and cytoplasmic compartments, though expression levels varied significantly by histotype and disease stage. For example, CCC displayed significantly lower LMTK3 expression in both compartments compared with other histotypes, particularly in early-stage disease, suggesting alternative regulatory pathways. Importantly, nuclear LMTK3 expression was significantly reduced in advanced-stage tumors relative to early-stage disease, while cytoplasmic levels remained stable. Despite this divergence, nuclear-cytoplasmic correlation remained strong across most histotypes and stages, indicating preserved co-regulation even in advanced disease. These patterns are consistent with studies in other cancers where nuclear and cytoplasmic LMTK3 expression has been associated with tumor grade, survival, and progression.^15^^,^^19^^,^^26^^,^^29^^,^^30^

Survival analyses further emphasized the importance of subcellular localization. High nuclear LMTK3 expression was confirmed to be associated with better prognosis in both OS and DSS context in early-stage EOC, reinforcing its proposed pro-tumorigenic role. In contrast, this association reversed in advanced-stage disease, where cytoplasmic dominance was indicative of improved survival. This stage-dependent switch in prognostic relevance suggests a functional transition, potentially reflecting a shift from proliferative to survival signaling roles. Mechanistically, this is supported by prior findings demonstrating that cytoplasmic LMTK3 promotes tumor cell survival in OC via interaction with integrin αV/β1 and inhibition of apoptotic signaling.^24^ Complementary studies in thyroid and bladder cancers have shown that LMTK3 knockdown induces apoptosis and cell-cycle arrest, suggesting a conserved survival-promoting role across malignancies.^19^^,^^21^ Additionally, in breast cancer, LMTK3 modulates ERα signaling in a compartment-specific manner, highlighting the spatial specificity of its oncogenic functions.^17^^,^^31^

Despite emerging functional evidence, LMTK3 remains a comparatively understudied (“dark”) kinase, and its broader regulatory networks in cancer are incompletely defined.^32^ To explore whether the pronounced stage- and compartment-specific protein behavior observed in our cohort was reflected at the transcriptomic level, we performed exploratory analyses using publicly available TCGA OC datasets. These analyses revealed only modest global co-expression patterns and failed to identify a dominant LMTK3-centered transcriptional program. Unbiased GO enrichment of the most strongly correlated transcripts pointed to broad processes related to membrane trafficking, vesicle dynamics, and cell adhesion rather than a single oncogenic signaling axis. Targeted correlation analyses with selected genes implicated in proliferation, apoptosis, and EMT showed weak but consistent associations, including positive correlations with integrin-related genes and proliferation markers, and weaker or inverse correlations with apoptotic mediators. Collectively, these findings suggest that LMTK3 function in OC may be regulated predominantly at the post-transcriptional or protein level and that bulk transcriptomic data provide limited resolution for capturing its spatially defined biological roles.^33^^,^^34^ Despite these insights, several limitations must be acknowledged. Subgroup analyses, especially in less common histotypes such as CCC and MC, were constrained by small sample sizes. To mitigate this problem in future studies, we are currently establishing an independent validation TMA cohort. Furthermore, we plan to implement compartment-specific protein analysis (e.g., subcellular fractionation and western blotting) and LMTK3-focused spatial omics approaches to uncover the molecular mechanisms underlying the observed compartmental dynamics. Finally, while transcriptomic analyses provided useful context, their exploratory nature and reliance on bulk tumor data limit mechanistic inference, underscoring the need for future studies integrating spatial proteomics and functional perturbation models to resolve compartment-specific LMTK3 signaling.

In conclusion, this study introduces LMTK3—and its subcellular localization—as a potential biomarker involved in epithelial ovarian tumor initiation, histotype-specific differentiation, and disease progression. Our findings reveal that spatial regulation of LMTK3 is dynamic, context-dependent, and prognostically relevant, particularly in early-stage disease. These data warrant further validation and functional studies to assess LMTK3’s utility for risk stratification, biological subtyping, and potentially therapeutic targeting in EOC.

## Methods

### TMA cohort

Formalin-fixed, paraffin-embedded (FFPE) tumor samples were retrieved for 532 patients diagnosed with EOC between 1993 and 2015 at Sahlgrenska University Hospital (Gothenburg, Sweden). Using triplicate 1-mm cores accounting for tumor heterogeneity, two TMA sets were constructed from (1) 204 early-stage (stage I–II) EOC cases^24^ and (2) 341 benign, borderline, and malignant EOC cases. In total, 350/532 malignant samples (stage I–IV) were evaluable and stratified into five histotypes (CCC, EC, HGSC, LGSC, and MC). Histological diagnoses were reviewed and updated by a board-certified pathologist in accordance with the 2016 World Health Organization classification guidelines. Clinicopathological information was retrieved from the Swedish Cancer Registry, the National Quality Registry, and the Cause of Death Registry. Herein, all malignant cases are referred to as the “complete EOC cohort,” which was further stratified, following FIGO guidelines, into “early-stage” (stage I–II) and “advanced-stage” (stage III–IV) subcohorts.

### IHC and scoring

IHC was performed to examine LMTK3 subcellular localization in EOC using a polyclonal antibody (ABIN5532611, 1:75 dilution). The control TMA set included 15 samples from four major histotypes (CCC, EC, HGSC, and MC) and corresponding FFPE control samples. Staining was performed using the Dako Autostainer Plus and EnVision FLEX visualization systems, including deparaffinization and antigen retrieval with EnVision FLEX High pH Target Retrieval Solution. Immunostaining used DAB (3,3′-Diaminobenzidine) and hematoxylin counterstaining. Sections were rinsed, dehydrated, cleared in xylene, and mounted. TMA slides were digitized at 20× magnification using an Olympus whole-slide scanner, and representative full-core images were extracted.

TMA sections were also stained with H&E for histological evaluation by an independent pathologist blinded to clinical data. Staining intensity and percentage of positively stained cells were assessed for nuclear and cytoplasmic compartments. A semi-quantitative H-score was calculated using the formula: [1 × (% of 1+) + 2 × (% of 2+) + 3 × (% of 3+)], yielding a total score from 0 to 300. Intensity was categorized as: 0 = negative, 1+ = weak positive, 2+ = moderate positive, and 3+ = strong positive. The highest H-score from the three cores was used as the representative LMTK3 expression for each tumor sample.

A categorical variable was defined to describe LMTK3 patterns as nuclear-dominant (N > C), balanced (N = C), or cytoplasmic-dominant (N < C). A continuous variable, termed the localization shift score (cytoplasmic minus nuclear H-score), quantified subcellular localization. This study applies the H-score method, which combines intensity and the proportion of positive cells, enabling more flexible cutoff determination for prognostic analyses.

### Statistical analysis

Statistical analyses were performed using R/Bioconductor (v4.4.0), with significance set at *p* < 0.05. Differences in LMTK3 H-scores across histotypes, stages, and compartments were assessed using the Kruskal-Wallis test, followed by pairwise Wilcoxon rank-sum tests with Benjamini-Hochberg correction. Associations between categorical variables and clinicopathological parameters were evaluated using Chi-squared and Fisher’s exact tests. Spearman correlation analysis was performed for nuclear and cytoplasmic LMTK3 H-scores. All statistical tests were conducted using base R (v4.4.0).

### Prognostic modeling based on LMTK3 expression and subcellular distribution

Patients were followed up through the Swedish Cancer Registry, the National Quality Registry, and the Cause of Death Registry until April 11, 2019. Survival data were calculated from the date of initial diagnosis to the date of death from any cause for OS or to the date of death from EOC for DSS. If the date of death was unavailable or the patient was lost to follow-up, data were censored on the last day of follow-up. To evaluate the prognostic relevance of LMTK3 expression, survival analyses were conducted based on both absolute H-scores and the relative subcellular distribution of expression. Nuclear and cytoplasmic H-scores were dichotomized into low and high expression groups using maximally selected rank statistics (surv_cutpoint tool, survminer package, v0.4.9) for both OS and DSS. Cut points were determined via survminer:surv_cutpoint() (v0.4.9) separately for the complete cohort and the early-stage (stage I–II) and advanced-stage (stage III–IV) subgroups. KM survival curves were generated (using ggplot [v3.5.2] and the survival package [v3.8-3]) for both the dichotomized and relative expression groups (N > C, N = C, and N < C, with “N > C” serving as the reference), with differences assessed using the log-rank test, including pairwise comparisons for the three-group analysis. Univariable and multivariable Cox proportional hazards models were fitted to assess associations with OS and DSS. Multivariable models were adjusted using nuclear and cytoplasmic LMTK3 expression (as appropriate), age at diagnosis, histotype, and tumor stage (disease stage was only included in the full-cohort models). In the stratified models for early- and advanced-stage disease, age group and histotype were included as covariates. An interaction model was constructed using Cox proportional hazards regression, including subcellular localization, dichotomized stage group (early vs. advanced), age category, and histotype, to evaluate whether the prognostic effect of LMTK3 localization varied by disease stage. Adjusted HRs, 95% CIs, and adjusted *p* values were reported.

### Internal validation analyses

To assess the robustness of prognostic findings, internal validation was performed using resampling approaches. The cohort was randomly divided into training and test subsets, and Cox proportional hazards models were re-estimated to evaluate the reproducibility of effect sizes. Bootstrap resampling (1,000 iterations) was additionally conducted to assess the stability of HR estimates for nuclear and cytoplasmic LMTK3 expression across disease stages. Predictive performance was evaluated using concordance indices and time-dependent receiver operating characteristic (ROC) curves.

### RNA expression and RNA-protein concordance analyses

A subset of early-stage EOC cases with available RNA-seq data was used to evaluate the relationship between LMTK3 transcript abundance and protein localization. Gene-level count data were normalized using trimmed mean of M-values (TMM) normalization and transformed to log counts per million (logCPM). LMTK3 expression was extracted using the Ensembl gene identifier ENSG00000142235. Associations between RNA expression and nuclear, cytoplasmic, and total protein H-scores were assessed using Spearman correlation. Cox proportional hazards models were used to evaluate the association between RNA expression and OS, with RNA levels modeled continuously per standard deviation increase.

### Exploratory transcriptomic analyses using publicly available TCGA data (GEPIA2 platform)

Exploratory transcriptomic analyses were conducted using the GEPIA2 platform (http://gepia2.cancer-pku.cn/#index), which integrates RNA-seq data from TCGA OC cohorts. Two complementary approaches were used.

First, hypothesis-guided Spearman correlation analyses were performed between LMTK3 expression and selected genes implicated in tumor progression, proliferation, apoptosis, and EMT. Genes were selected a priori based on established roles in cancer biology and included integrin-related genes (ITGAV, ITGB1), proliferation markers (MKI67, CCNB1), apoptotic regulators (CASP3, CASP9, BAX, BCL2), and EMT-associated genes (SNAI1, VIM).

Second, an unbiased co-expression analysis was performed using GEPIA2 to identify transcripts most strongly correlated with LMTK3 expression. GO enrichment analysis was subsequently conducted on the top-correlated transcripts to identify overrepresented biological processes and molecular functions. In addition, LMTK3 mRNA expression levels were assessed across clinical stage groups using GEPIA2 to evaluate potential stage-dependent differences at the transcriptomic level.

## Data and code availability

All data were stored on a computer at the corresponding author’s main laboratory and can be requested from the corresponding author.

## Acknowledgments

We would like to express our gratitude to our former colleague Daniella Pettersson for her support and advice in the execution of the IHC procedures.

This study was performed in accordance with the Declaration of Helsinki, and the use of tumor material was approved by the Regional Ethical Review Board, which granted a waiver of written consent (Gothenburg, Sweden; registration number 767-14; Dnr 2025-01299-02).

This work has received funding from the 10.13039/501100002794Swedish Cancer Society (23 2732 Pj 01 H), the King Gustav V Jubilee Clinic Cancer Research Foundation (2022:410), and the LUA/ALF-agreement in West of Sweden health care region (ALFGBG-1005885).

## Author contributions

K.H., A.K., T.Z.P., and G.S. contributed to the conception and design of the study. H.S., E.I., and L.W. oversaw and managed the project design, ensuring its execution, with guidance from K.H., T.Z.P., P.K. K.S., A.L., and K.H. provided the supply of patient-derived raw material. E.I. performed the wet-lab experiments, while A.K. quantified the IHC. E.I. performed the primary data analysis, with assistance from K.H., H.S., L.W., P.K., and T.Z.P. in statistical analysis and interpretation. E.I. wrote the first draft of the manuscript, while K.H., T.Z.P., A.K., A.L., G.S., K.S., and P.K. contributed to revising the manuscript, refining the interpretation of the results, and enhancing the overall presentation. All authors read and approved the final manuscript, ensuring the accuracy of the content and conclusions.

## Declaration of interests

The authors have no relevant financial or non-financial interests to disclose.

## Declaration of generative AI and AI-assisted technologies in the writing process

During the preparation of this work, the author(s) used OpenAI to assist with coding for data processing and to support language enhancement during the drafting phase of the manuscript. After using this tool or service, the author(s) reviewed and edited the content as needed and take(s) full responsibility for the content of the publication.

## Supplemental information

Document S1. Figures S1–S5 and Tables S1–S3

Document S2. Article plus supplemental information
