# Supplementary material for: LMTK3 as a spatially regulated and stage-dependent biomarker in epithelial ovarian tumorigenesis
Source: Mol Ther Oncol. 2026 Apr 6;34(2):201199. doi: 10.1016/j.omton.2026.201199 (PMC13123347; doi:10.1016/j.omton.2026.201199)
Supplement: Document S1. Figures S1–S5 and Tables S1–S3 [file mmc1.pdf]

OMTON, Volume *34*

Supplemental information

LMTK3 as a spatially regulated and stage-dependent biomarker in epithelial ovarian tumorigenesis

Ella Ittner, Lucas Werner, Hugo Swenson, Anna Linder, Karin Sundfeldt, Ghassan Saed, Per Karlsson, Toshima Z. Parris, Anikó Kovács, and Khalil Helou

Supplemental Information

Table S1 - Clinical, pathological, and LMTK3 expression characteristics of complete cohort stratified by ovarian tumor type

Continuous variables (indicated via *) were compared using the Wilcoxon rank-sum test. Categorical variables were compared using Chi-squared or Fisher's Exact Test, as appropriate. Abbreviations: CCC: clear cell carcinoma, EC: endometrioid carcinoma, EOC: Epithelial-ovarian cancer, HGSC: high-grade serous carcinoma, LGSC: low-grade serous carcinoma, LMTK3: Lemur-Tyrosine-Kinase 3, MC: mucinous carcinoma, TMA: Tissue micro array.

	MA-based study cohort across the EOC tumor spectru				
	Overall	benign	borderline type	malignant	
	532	123	59	350	
**Age at Diagnosis (median [IQR])**	62.00 [52.00, 72.00]	61.00 [50.50, 71.00]	52.00 [39.00, 63.50]	63.00 [55.00, 73.00]	<0.001*
**Menopausal status**					
Premenopausal (<50 years)	100 (18.8)	28 (22.8)	26 (44.1)	46 (13.1)	<0.001
Early postmenopausal (50-59 years)	131 (24.6)	25 (20.3)	15 (25.4)	91 (26.0)	
Intermediate postmenopausal (60-69 years)	144 (27.1)	35 (28.5)	10 (16.9)	99 (28.3)	
Late postmenopausal (≤70 years)	157 (29.5)	35 (28.5)	8 (13.6)	114 (32.6)	
**Stage**					
I	181 (34.0)	0 (0.0)	49 (83.1)	132 (37.7)	<0.001
II	68 (12.8)	0 (0.0)	1 (1.7)	67 (19.1)	
III	142 (26.7)	0 (0.0)	9 (15.3)	133 (38.0)	
IV	18 (3.4)	0 (0.0)	0 (0.0)	18 (5.1)	
Not applicable	123 (23.1)	123 (100.0)	0 (0.0)	0 (0.0)	
**Histotype (%)**					
CCC	42 (7.9)	0 (0.0)	0 (0.0)	42 (12.0)	<0.001
EC	51 (9.6)	0 (0.0)	0 (0.0)	51 (14.6)	
HGSC	209 (39.3)	0 (0.0)	0 (0.0)	209 (59.7)	
LGSC	18 (3.4)	0 (0.0)	0 (0.0)	18 (5.1)	
MC	30 (5.6)	0 (0.0)	0 (0.0)	30 (8.6)	
Not applicable	182 (34.2)	123 (100.0)	59 (100.0)	0 (0.0)	
**Nuclear staining intensity**					
Negative	3 (0.6)	0 (0.0)	0 (0.0)	3 (0.9)	<0.001
Low	53 (10.0)	8 (6.5)	5 (8.5)	40 (11.4)	
Moderate	105 (19.7)	13 (10.6)	19 (32.2)	73 (20.9)	
High	358 (67.3)	89 (72.4)	35 (59.3)	234 (66.9)	
Not available	13 (2.4)	13 (10.6)	0 (0.0)	0 (0.0)	
**Nuclear positivity (% of cells)**					
None (0%)	3 (0.6)	0 (0.0)	0 (0.0)	3 (0.9)	<0.001
Low (1-10%)	7 (1.3)	2 (1.6)	1 (1.7)	4 (1.1)	
Moderate (11-50%)	21 (3.9)	1 (0.8)	1 (1.7)	19 (5.4)	
High (51-100%)	488 (91.7)	107 (87.0)	57 (96.6)	324 (92.6)	
Not available	13 (2.4)	13 (10.6)	0 (0.0)	0 (0.0)	
**Nuclear H-score (median [IQR])**	300.00 [200.00, 300.00]	300.00 [240.00, 300.00]	270.00 [180.00, 300.00]	300.00 [185.00, 300.00]	0.052 *
**Cytoplasmic staining intensity**					
Negative	6 (1.1)	3 (2.4)	0 (0.0)	3 (0.9)	<0.001
Low	57 (10.7)	2 (1.6)	11 (18.6)	44 (12.6)	
Moderate	143 (26.9)	11 (8.9)	19 (32.2)	113 (32.3)	
High	257 (48.3)	56 (45.5)	16 (27.1)	185 (52.9)	
Not available	69 (13.0)	51 (41.5)	13 (22.0)	5 (1.4)	
**Cytoplasmic positivity (% of cells)**					
None (0%)	6 (1.1)	3 (2.4)	0 (0.0)	3 (0.9)	<0.001
Moderate (11-50%)	6 (1.1)	0 (0.0)	2 (3.4)	4 (1.1)	
High (51-100%)	451 (84.8)	69 (56.1)	44 (74.6)	338 (96.6)	
Not available	69 (13.0)	51 (41.5)	13 (22.0)	5 (1.4)	
**Cytoplasmic H-score (median [IQR])**	270.00 [200.00, 300.00]	300.00 [200.00, 300.00]	200.00 [115.00, 300.00]	300.00 [200.00, 300.00]	0.012 *
**LMTK3 localization pattern**					
Nuclear-dominant (N > C)	119 (22.4)	35 (28.5)	17 (28.8)	67 (19.1)	<0.001
balanced (C = N)	298 (56.0)	60 (48.8)	27 (45.8)	211 (60.3)	
Cytoplasmic-dominant (C > N)	79 (14.8)	9 (7.3)	2 (3.4)	68 (19.4)	
Not available	36 (6.8)	19 (15.4)	13 (22.0)	4 (1.1)	

Table S2 - Compiled survival statistics of all performed survival modeling including statistics of univariate, multivariate cox regression and KM logrank-test evaluating the prognostic effect of LMTK3 expression in EOC

Abbreviations: CI: Confidence interval, DSS: Disease-specific survival, EOC: epithelial ovarian cancer, HR: Hazard ratio, LMTK3: Lemur Tyrosine Kinase 3, KM: Kaplan–Meier, multi: multivariate cox model, OS: Overall survival, uni: univariate cox model.

Survival Type	Survival Model	Uni HR	Uni 95% CI	Uni p	Multi HR	Multi 95% CI	Multi p	Log-rank p
OS	Complete - Nuclear	0.65	0.48-0.89	p = 0.006	0.68	0.5-0.93	p = 0.016	p = 0.006
OS	Complete - Cytoplasmic	0.89	0.61-1.29	p = 0.523	0.93	0.63-1.36	p = 0.701	p = 0.523
OS	Early - Nuclear	0.35	0.22-0.56	p < 0.001	0.33	0.2-0.54	p < 0.001	p < 0.001
OS	Early - Cytoplasmic	0.68	0.41-1.13	p = 0.132	0.79	0.45-1.38	p = 0.412	p = 0.129
OS	Late - Nuclear	1.48	0.98-2.24	p = 0.065	1.5	0.98-2.29	p = 0.063	p = 0.063
OS	Late - Cytoplasmic	1.34	0.79-2.26	p = 0.272	1.05	0.61-1.82	p = 0.860	p = 0.270
DSS	Complete - Nuclear	0.6	0.36-0.99	p = 0.045	0.63	0.38-1.06	p = 0.081	p = 0.043
DSS	Complete - Cytoplasmic	0.9	0.55-1.46	p = 0.665	0.99	0.61-1.62	p = 0.969	p = 0.664
DSS	Early - Nuclear	0.33	0.18-0.59	p < 0.001	0.34	0.19-0.63	p < 0.001	p < 0.001
DSS	Early - Cytoplasmic	0.5	0.27-0.9	p = 0.021	0.62	0.33-1.19	p = 0.152	p = 0.019
DSS	Late - Nuclear	2.25	1.17-4.33	p = 0.015	2.55	1.26-5.14	p = 0.008	p = 0.013
DSS	Late - Cytoplasmic	3.25	1-10.56	p = 0.05	2.42	0.72-8.15	p = 0.154	p = 0.038

Table S3.

Matched early-stage epithelial ovarian cancer dataset used for RNA–protein correlation analyses and RNA-based survival modeling.

The table includes pseudonymized sample identifiers, LMTK3 mRNA expression (logCPM), raw RNA-seq counts, nuclear and cytoplasmic LMTK3 H-scores, total LMTK3 expression, and overall survival annotations (OS_days, OS_status). Sample identifiers were pseudonymized to prevent patient re-identification.

Public_ID	OS_days	OS_status	LMTK3_logCP	LMTK3_rawC	Hscore_1hit_I	Hscore_1hit_(	Hscore_total
EOC_1	4620	1	1.42647454	18	300	300	600
EOC_2	2198	1	1.68165911	30	160	300	460
EOC_3	1348	1	-3.09472966	0	300	300	600
EOC_4	793	1	-3.09472966	0	300	300	600
EOC_5	1129	1	-3.09472966	0	300	300	600
EOC_6	4876	1	1.74002349	31	300	300	600
EOC_7	1637	1	2.96776935	69	60	300	360
EOC_8	3813	1	1.63906266	32	160	100	260
EOC_9	3486	1	0.73353981	13	300	300	600
EOC_10	2815	1	-2.08490722	1	300	300	600
EOC_11	3303	1	3.81026449	129	300	300	600
EOC_12	1844	1	2.95398093	67	300	300	600
EOC_13	3266	1	-2.18633301	1	300	300	600
EOC_14	1731	1	-3.09472966	0	80	200	280
EOC_15	3466	1	-2.12442252	1	300	300	600
EOC_16	3946	1	1.03004858	19	300	300	600
EOC_17	2925	1	3.1614759	73	300	300	600
EOC_18	4721	1	0.8862246	13	90	100	190
EOC_19	1355	1	-2.02020279	1	300	300	600
EOC_20	2072	1	-2.29935038	1	200	300	500
EOC_21	5576	0	2.88312248	65	200	200	400
EOC_22	2793	1	2.78561478	45	300	300	600
EOC_23	1001	1	2.96755093	71	200	200	400
EOC_24	366	1	0.83723688	14	40	200	240
EOC_25	5424	0	-2.08562808	1	300	300	600
EOC_26	3516	1	0.85046406	14	80	100	180
EOC_27	1330	1	-3.09472966	0	300	300	600
EOC_28	4548	1	-3.09472966	0	300	300	600
EOC_29	1891	1	1.01290468	20	300	300	600
EOC_30	2242	1	-3.09472966	0	300	200	500
EOC_31	3453	1	1.92340116	32	300	300	600
EOC_32	1543	1	1.01003586	19	300	300	600
EOC_33	5201	0	3.71431886	117	270	270	540
EOC_34	5211	0	1.61724259	25	300	300	600
EOC_35	5182	0	2.90060361	78	300	300	600
EOC_36	5168	0	-2.10953974	1	300	300	600
EOC_37	4604	1	-3.09472966	0	300	300	600
EOC_38	5098	0	1.79390012	37	300	200	500
EOC_39	5065	0	1.41757596	26	300	300	600
EOC_40	5005	0	0.59708068	13	300	120	420
EOC_41	4994	0	1.26562644	19	300	300	600
EOC_42	4917	1	1.59675834	19	200	200	400
EOC_43	239	1	1.44114509	26	200	200	400
EOC_44	1113	1	1.69843667	30	300	300	600
EOC_45	3235	1	-3.09472966	0	200	200	400
EOC_46	3994	1	2.82559126	52	200	20	220
EOC_47	1657	1	-3.09472966	0	300	300	600
EOC_48	3436	1	1.66299652	25	200	200	400
EOC_49	1440	1	1.98965293	36	300	200	500
EOC_50	1697	1	-2.22352434	1	300	200	500
EOC_51	731	1	2.98579031	74	80	200	280
EOC_52	6473	1	0.93475505	13	100	200	300
EOC_53	1898	1	2.08746576	41	300	300	600
EOC_54	226	1	2.51533617	53	100	200	300
EOC_55	590	1	2.32584931	43	300	300	600
EOC_56	665	1	-2.27862464	1	300	300	600
EOC_57	3064	1	1.62418462	25	200	200	400
EOC_58	1899	1	-3.09472966	0	70	0	70
EOC_59	1894	1	3.46607734	88	200	100	300
EOC_60	1505	1	0.62336281	14	180	300	480
EOC_61	1997	1	-3.09472966	0	300	300	600
EOC_62	3314	1	-1.76722449	1	300	300	600
EOC_63	2179	1	1.05297676	13	300	300	600
EOC_64	1779	1	-3.09472966	0	180	200	380
EOC_65	1454	1	-3.09472966	0	300	300	600
EOC_66	2391	1	-1.93489265	1	300	300	600
EOC_67	1192	1	2.9197629	60	300	300	600
EOC_68	2039	1	-2.17289961	1	200	200	400
EOC_69	1485	1	2.71287358	51	10	100	110
EOC_70	1023	1	-3.09472966	0	140	200	340
EOC_71	927	1	-3.09472966	0	300	300	600
EOC_72	607	1	1.64818936	25	60	90	150
EOC_73	1758	1	0.6223735	13	300	300	600
EOC_74	958	1	2.77446897	47	300	300	600
EOC_75	5335	1	1.86718426	30	300	200	500
EOC_76	1187	1	-2.12045486	1	300	300	600
EOC_77	4372	1	-3.09472966	0	0	100	100
EOC_78	492	1	-3.09472966	0	300	300	600

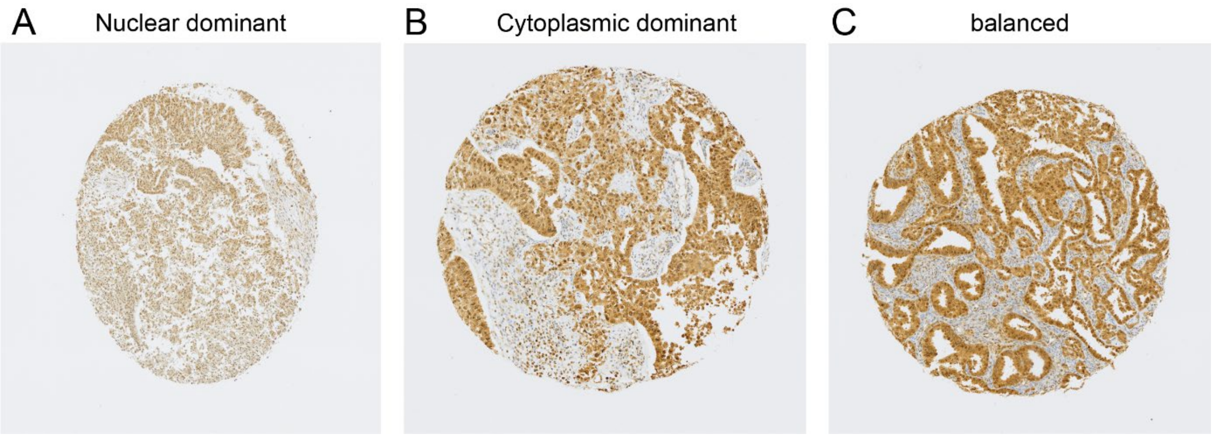

Figure S1. Representative immunohistochemical staining patterns of LMTK3 in early-stage HGSC.

Representative images illustrate (A) nuclear-dominant, (B) cytoplasmic-dominant, and (C) balanced nuclear and cytoplasmic LMTK3 expression in early-stage HGSC samples. Images represent full tissue microarray cores extracted from whole-slide scans acquired at 20× magnification. HGSC: high-grade serous carcinoma, LMTK3: Lemur-Tyrosine-Kinase 3

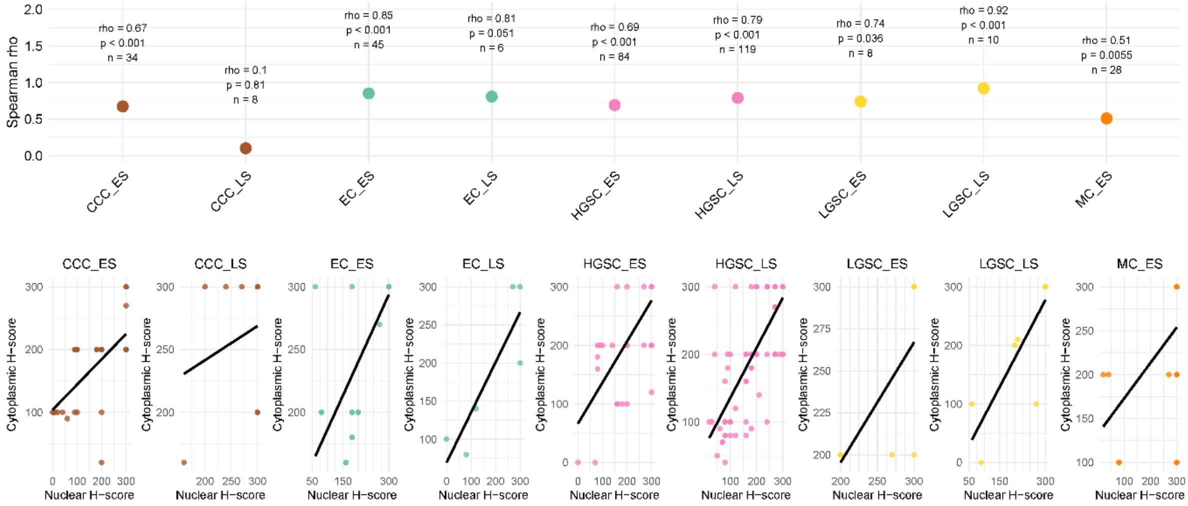

Figure S2. Correlation between nuclear and cytoplasmic LMTK3 expression across the malignant EOC subcohort stratified by stage and histotype

(A) Spearman correlation coefficients (ρ) between nuclear and cytoplasmic LMTK3 H-scores across histotypes stratified into early-stage (ES) and advanced-stage (LS) subsets. Boxplots summarize the correlation strength; individual dots represent the observed correlations, with annotated ρ, p-value, and sample size (n) for each group. Scatterplots below visualize the subcellular correlation for each group. Linear regression lines are shown in black. Abbreviations: CCC: clear cell carcinoma, EC: endometrioid carcinoma, EOC: Epithelial-ovarian cancer, ES: early-stage, HGSC: high-grade serous carcinoma, LGSC: low-grade serous carcinoma, LMTK3: Lemur-Tyrosine-Kinase 3, LS: late-stage/advanced-stage, MC: mucinous carcinoma.

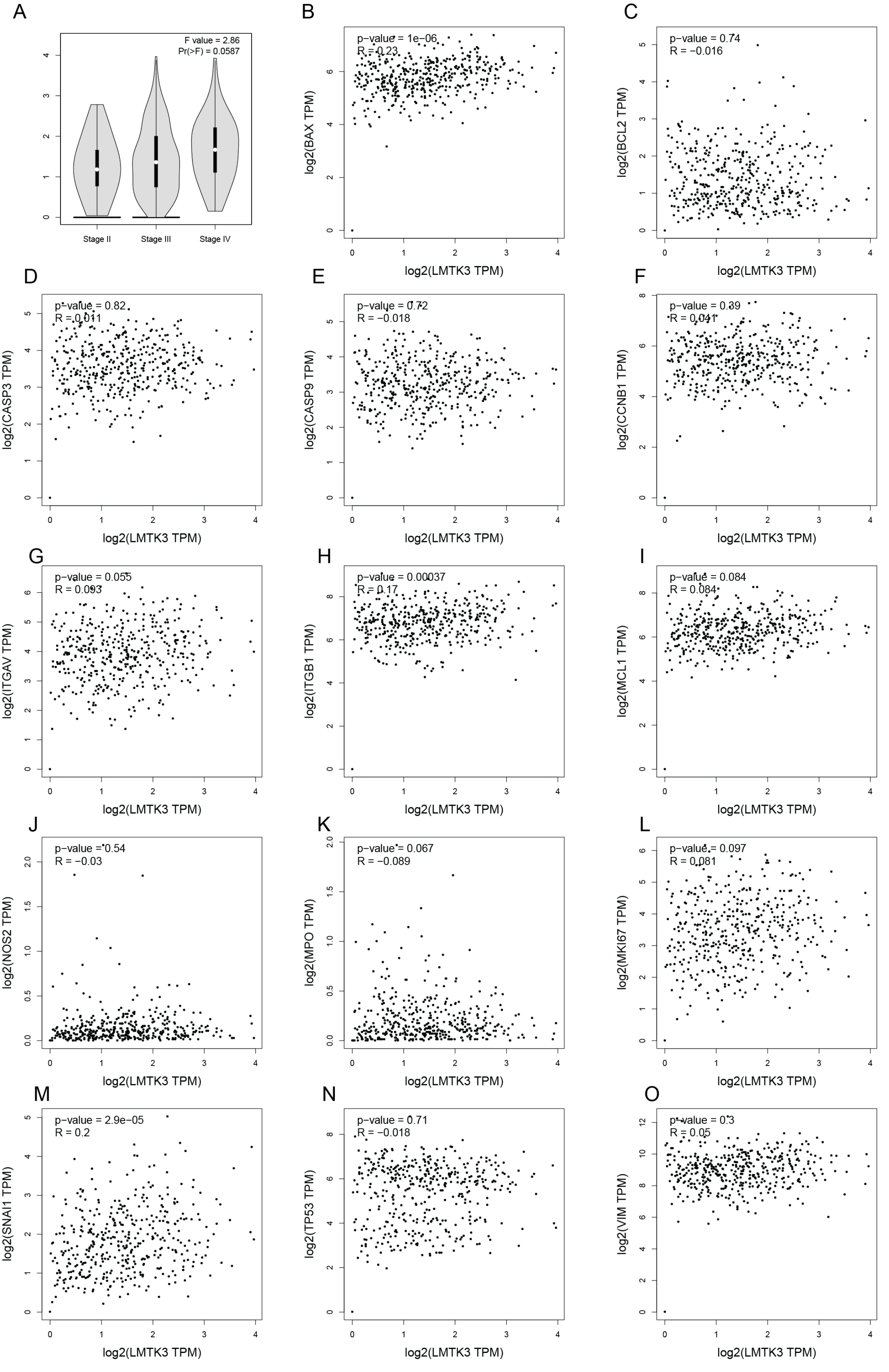
A
F value = 2.86
Pr(>F) = 0.0587
Stage II
Stage III
Stage IV
B
p−value = 1e−06
R = 0.23
log2(BAX TPM)
C
p−value = 0.74
R = −0.016
log2(BCL2 TPM)
D
p−value = 0.82
R = 0.011
log2(CASP3 TPM)
E
p−value = 0.72
R = −0.018
log2(CASP9 TPM)
F
p−value = 0.39
R = 0.041
log2(CCNB1 TPM)
G
p−value = 0.055
R = 0.093
log2(ITGAV TPM)
H
p−value = 0.00037
R = 0.17
log2(ITGB1 TPM)
I
p−value = 0.084
R = 0.084
log2(MCL1 TPM)
J
p−value = 0.54
R = −0.03
log2(NOS2 TPM)
K
p−value = 0.067
R = −0.089
log2(MPO TPM)
L
p−value = 0.097
R = 0.081
log2(MKI67 TPM)
M
p−value = 2.9e−05
R = 0.2
log2(SNAI1 TPM)
N
p−value = 0.71
R = −0.018
log2(TP53 TPM)
O
p−value = 0.3
R = 0.05
log2(VIM TPM)
log2(LMTK3 TPM)

Figure S3. Exploratory transcriptomic analyses of LMTK3 in TCGA ovarian cancer.

(A) LMTK3 mRNA expression across clinical stage groups in TCGA ovarian cancer (TCGA-OV) as assessed using the GEPIA2 platform. No statistically significant differences were observed between stages (ANOVA p = 0.0587). (B–O) Spearman correlation analyses between LMTK3 mRNA expression and selected genes implicated in apoptosis (BAX, BCL2, CASP3, CASP9), proliferation (CCNB1, MKI67), adhesion and integrin signaling (ITGAV, ITGB1), epithelial–mesenchymal transition (SNAI1, MCL1), and inflammatory or stress-related pathways (NOS2, MPO, TP53, VIM). Gene expression values are shown as log2-transformed TPM. Correlation coefficients (R) and p-values are indicated in each panel. Overall, correlations were modest, and no dominant transcriptional program associated with LMTK3 expression was identified.

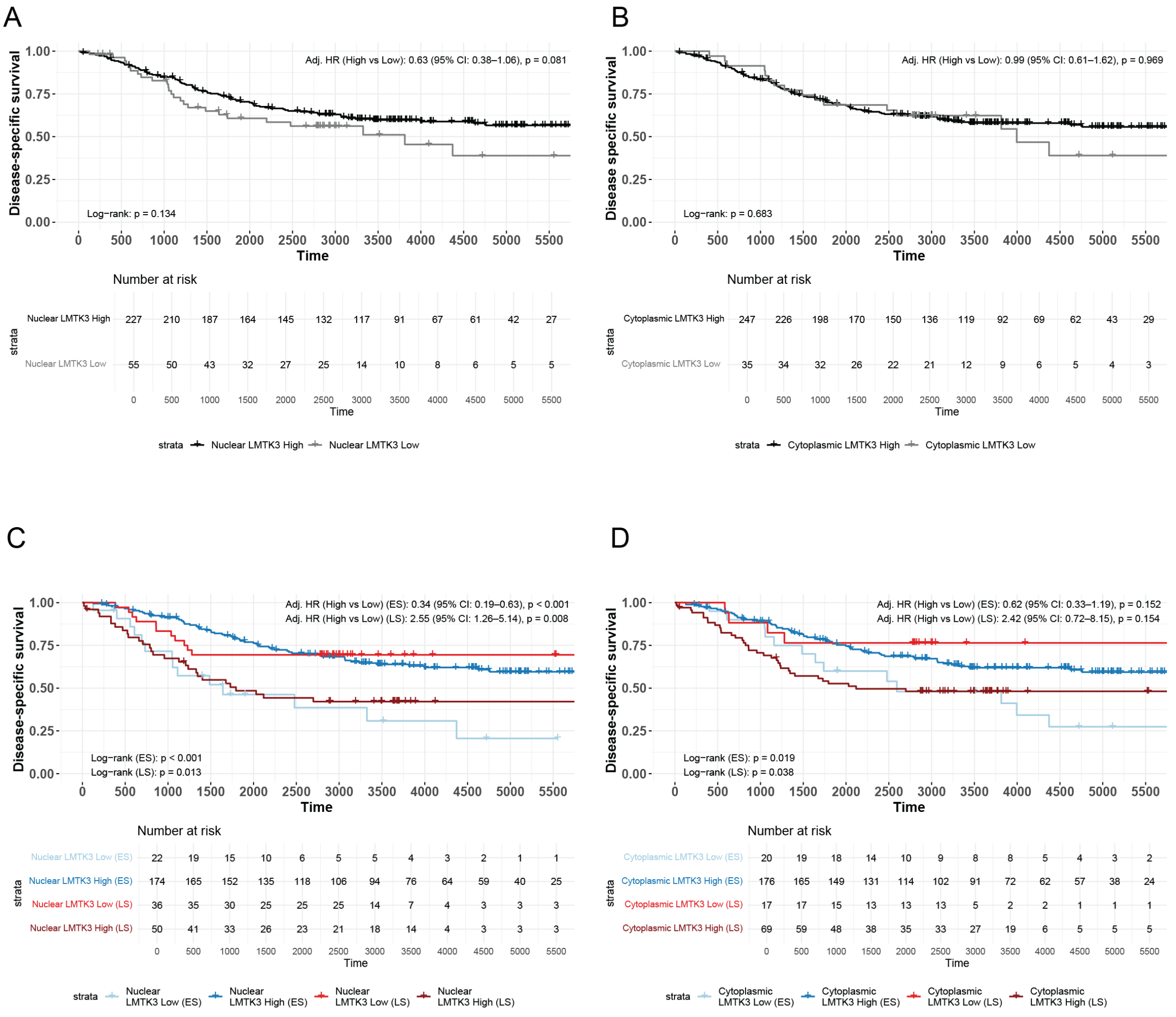

Figure S4 Disease-specific survival impact of absolute LMTK3 expression in nucleus and cytoplasm across complete, early-, and advanced-stage EOC

Kaplan–Meier disease-specific survival (DSS) curves, with a 15-year truncation, are shown for high vs. low absolute LMTK3 expression, based on optimized H-score cutoffs. (A) Nuclear expression across the complete EOC cohort. (B) Cytoplasmic expression in the same cohort. (C) Nuclear expression stratified by early-stage (Stage I/II) and advanced-stage (Stage III/IV) disease. (D) Cytoplasmic expression stratified by disease stage. Hazard ratios (HR) and adjusted p-values from multivariable Cox regression models (adjusted for age group, histotype, and stage where appropriate) are displayed within each panel. Statistical differences between survival curves were evaluated using log-rank testing also indicated in each subplot. Abbreviations: EOC: Epithelial-ovarian cancer, LMTK3: Lemur-Tyrosine-Kinase 3.

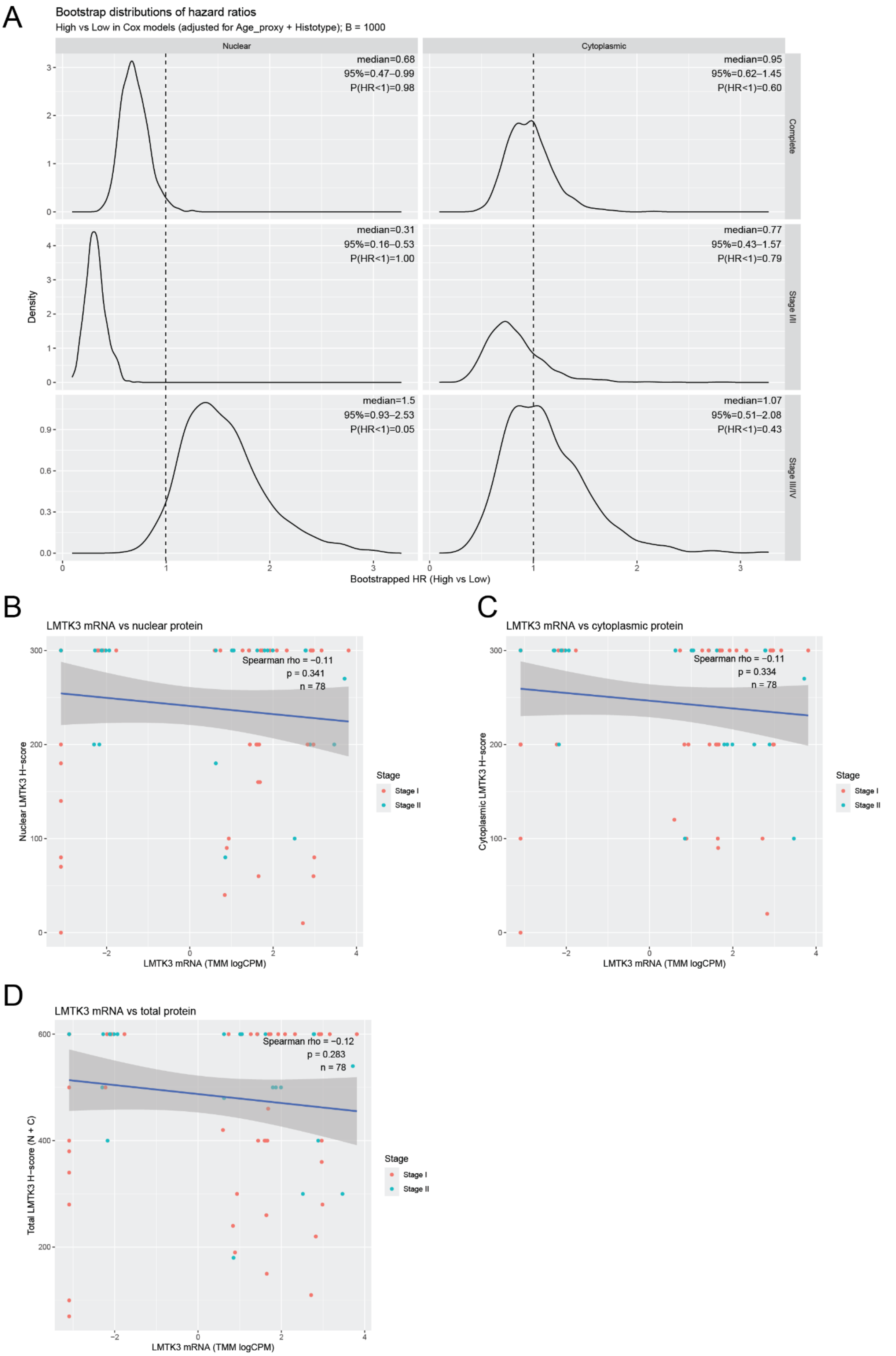
A
Bootstrap distributions of hazard ratios
High vs Low in Cox models (adjusted for Age_proxy + Histotype); B = 1000
Nuclear
Cytoplasmic
Complete
Stage I/II
Stage III/IV
median=0.68
95%=0.47–0.99
P(HR<1)=0.98
median=0.95
95%=0.62–1.45
P(HR<1)=0.60
median=0.31
95%=0.16–0.53
P(HR<1)=1.00
median=0.77
95%=0.43–1.57
P(HR<1)=0.79
median=1.5
95%=0.93–2.53
P(HR<1)=0.05
median=1.07
95%=0.51–2.08
P(HR<1)=0.43
Density
Bootstrapped HR (High vs Low)
B
LMTK3 mRNA vs nuclear protein
Spearman rho = −0.11
p = 0.341
n = 78
Nuclear LMTK3 H−score
LMTK3 mRNA (TMM logCPM)
Stage
Stage I
Stage II
C
LMTK3 mRNA vs cytoplasmic protein
Spearman rho = −0.11
p = 0.334
n = 78
Cytoplasmic LMTK3 H−score
LMTK3 mRNA (TMM logCPM)
Stage
Stage I
Stage II
D
LMTK3 mRNA vs total protein
Spearman rho = −0.12
p = 0.283
n = 78
Total LMTK3 H−score (N + C)
LMTK3 mRNA (TMM logCPM)
Stage
Stage I
Stage II

Figure S5. Internal validation of LMTK3 prognostic effects and RNA-protein concordance analyses.

(A) Bootstrap distributions of HRs for high versus low LMTK3 expression derived from Cox proportional hazards models stratified by subcellular localization (nuclear vs cytoplasmic) and disease stage (early vs advanced). Density plots summarize 1,000 bootstrap iterations, with dashed vertical lines indicating HR = 1. Median HRs, 95% confidence intervals, and proportions of bootstrap estimates exceeding HR = 1 are shown, demonstrating the stability and stage-dependent reversal of nuclear LMTK3 prognostic effects.

(B–D) Correlation analyses between LMTK3 mRNA expression (logCPM) and nuclear (B), cytoplasmic (C), and total (D) LMTK3 total protein H-scores (nucleic + cytoplasmic expression) in matched early-stage EOC cases. Points are colored by disease stage. Spearman correlation coefficients (ρ) and p-values are indicated. Overall, RNA–protein correlations were weak and not statistically significant, supporting limited concordance between transcript abundance and protein localization. Abbreviations: HR: Hazard ratio, LMTK3: Lemur-Tyrosine-Kinase 3.
